# Pan-Cancer Identification of Prognostic-Associated Metabolic Pathways

**DOI:** 10.3390/biology12081129

**Published:** 2023-08-14

**Authors:** Wenbo Chen, Xin Chen, Zhenyu Zhao, Menglu Li, Shuang Dong, Sheng Hu, Xiaoyu Li, Mingqian Feng, Ke Chen, Shan Zhong, Chunjiang He

**Affiliations:** 1 School of Basic Medical Sciences, Wuhan University, Wuhan 430071, China; 2 College of Biomedicine and Health, Huazhong Agricultural University, Wuhan 430070, China; 3 College of Life Science and Technology, Huazhong Agricultural University, Wuhan 430070, China; 4 Department of Urology, Tongji Hospital, Tongji Medical College, Huazhong University of Science and Technology, Wuhan 430070, China; 5 College of Informatics, Huazhong Agricultural University, Wuhan 430070, China; 6 Department of Oncology, Hubei Cancer Hospital, Tongji Medical College, Huazhong University of Science and Technology, Wuhan 430079, China

**Keywords:** cancer transcriptome, metabolic pathways, prognosis

## Abstract

**Simple Summary:**

When studying the cancer transcriptome, we found that there is no work that has conducted a global and systematic analysis of the metabolism-related genes and pathways in the cancer transcriptome, especially in connection with cancer prognosis and treatment. Therefore, we performed this work by establishing a survival risk model to associate metabolic checkpoint genes and pathways in cancer with prognosis. We screened metabolic genes and pathway signatures related to cancer survival, providing data reference for clinical treatment.

**Abstract:**

Metabolic dysregulation has been reported involving in the clinical outcomes of multiple cancers. However, systematical identification of the impact of metabolic pathways on cancer prognosis is still lacking. Here, we performed a pan-cancer analysis of popular metabolic checkpoint genes and pathways with cancer prognosis by integrating information of clinical survival with gene expression and pathway activity in multiple cancer patients. By discarding the effects of age and sex, we revealed extensive and significant associations between the survival of cancer patients and the expression of metabolic checkpoint genes, as well as the activities of three primary metabolic pathways: amino acid metabolism, carbohydrate metabolism, lipid metabolism, and eight nonprimary metabolic pathways. Among multiple cancers, we found the survival of kidney renal clear cell carcinoma and low-grade glioma exhibit high metabolic dependence. Our work systematically assesses the impact of metabolic checkpoint genes and pathways on cancer prognosis, providing clues for further study of cancer diagnosis and therapy.

## 1. Introduction

Metabolic genes and pathways of tumor cells have been widely recognized as an emerging hallmark of cancer, and metabolic rewiring is critical for the initiation, proliferation, and progression of cancer [1]. Metabolic checkpoints play a crucial role in dynamically coordinating metabolic homeostasis in the tumor microenvironment, referring to a series of cellular metabolic molecular switches that consist of metabolic signals, sensors, signal transducers, and molecular effectors [2]. Metabolic checkpoints are involved in a molecular mechanism that senses metabolic stress caused by nutrient deprivation [3]. Metabolic checkpoints are also inherently linked to the development, activation, function, differentiation, and survival of T cells [4]. For example, the expression of HIF-1α (HIF1A) is critical for the response of T cells to hypoxic environments [5]. The activity of intracellular metabolic pathways is closely related to the unique metabolic homeostasis in the tumor microenvironment [6]. Including glucose and lactate, tumors can use a variety of fuels to obtain energy to sustain their survival, such as fatty acids, amino acids, and proteins [7]. Deprivation of glutamine impairs the activation-induced growth and proliferation of T cells [8]. Meanwhile, the regulation of cell metabolic pathways against the lifespan and anticancer function of tumor T cells directly affects the clinical efficacy of T cell-mediated immunotherapy [9]. Despite the importance of metabolic checkpoints and pathways, our current understanding of how the metabolic network affects the progression of cancers is still incomplete [10]. Thus, a systematic dissection of their relevance to cancer treatment and prognosis is necessary.

Here, we integrated transcriptomic and clinical data from 33 cancer types in TCGA and constructed a multifactor cox regression model to assess the impact of metabolic checkpoint gene expression and pathway activities on patient survival. Our study comprehensively explored the association between the dysregulation of metabolic transcriptome and cancer prognosis, providing a clue for finding new cancer therapeutic markers and selecting effective treatments.

## 2. Materials and Methods

### 2.1. Data Collection

Gene expression data from cancer patients in the TCGA cohort were obtained from UCSC Xena (http://xena.ucsc.edu/, accessed on 23 September 2022) [11]. Expression value was represented by log_2_ (x + 1), while x indicates the RSEM normalized counts of each gene. Clinical data of the samples, including sample barcodes, age, gender, survival outcome, overall survival, etc., were obtained from GDC (https://portal.gdc.cancer.gov/, accessed on 25 September 2022). The sample information from the two databases was matched using the sample barcodes. Samples with unclear survival outcomes or survival time were excluded. All data were publicly available.

### 2.2. Collection of Metabolic Checkpoint Genes

Twenty-six metabolic checkpoint genes were collected from previous work [12]. Protein–protein interaction analysis was performed using the online analysis platform STRING (https://string-db.org/, accessed on 5 January 2023). The list of metabolic checkpoint genes was used as input. The protein products of all 26 metabolic checkpoint genes were correctly matched, and a protein–protein interaction network was generated. The generated network was exported to Cytoscape for network visualization. In the protein–protein interaction network diagram, each line between two proteins represents an interaction between them.

### 2.3. Calculation of Metabolic Pathway Activity Scores

Metabolic pathways were obtained from the public platform KEGG (https://www.kegg.jp/, accessed on 8 October 2022) [13], which included 84 metabolic pathways from 11 major metabolic categories. Based on the gene expression data in each sample and the genes in each gene set, the ssGSEA score of each pathway was calculated using the R package GSVA v1.46.0 [14], reflecting the enrichment level of the pathway in the sample.

### 2.4. Multivariable Cox Regression Model

According to the clinical information of the samples provided by GDC, the method of calculating the survival time (days_to_last_followup or days_to_death) was determined based on their survival status. In each cancer type, a multivariable Cox regression model was constructed using gene expression or metabolic pathway scores, age, and gender as independent variables. This analysis was performed using the R package survival v3.5. The hazard ratio (HR) output by the model reflects the degree of impact of the variable on prognosis. HR > 1 indicates that the variable increases the patient’s risk of death and leads to a poor prognosis. HR < 1 indicates that the variable reduces the patient’s risk of death and leads to a good prognosis. The significance threshold was set at the commonly used threshold, the Wald test *p*-value of the regression model was less than 0.05, and the Wald test *p*-value of the variable (gene expression or metabolic pathway score) was less than 0.05. When the significance threshold was met, we considered that gene expression or pathway scores had a significant impact on prognosis.

### 2.5. Statistical Analysis and Visualization

All statistical analyses were performed using R v4.2.0. Radar plots were drawn using the R package fmsb v0.7.5, and heat maps were drawn using the R package pheatmap v1.0.12. Other graphics were drawn using the R package ggpubr v0.4.0.

## 3. Results

### 3.1. Significant Correlation between the Pan-Cancer Survival and Metabolism Checkpoint Genes

**Figure 1:**
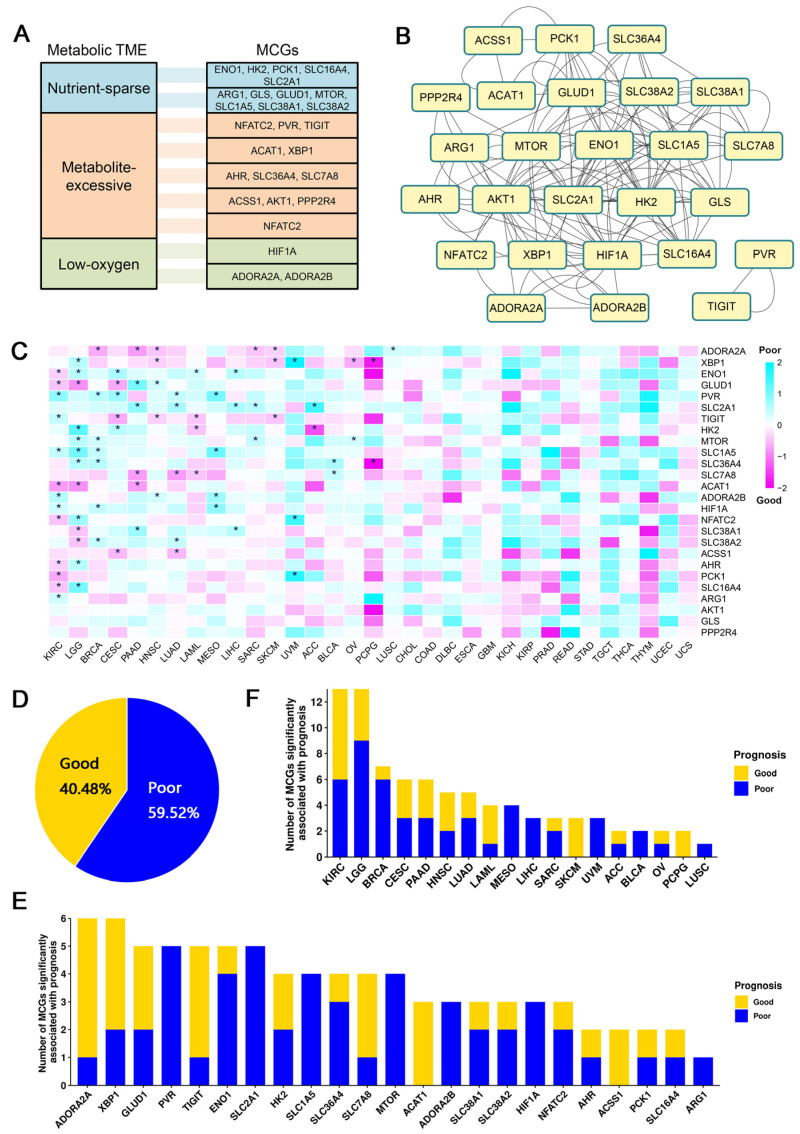
Global identification of survival-related metabolic checkpoint genes. (**A**) Twenty-six metabolic checkpoint genes were classified based on their functional role in the tumor microenvironment. (**B**) Interacting network diagram of metabolic checkpoint genes, where the lines represent interactions between genes. (**C**) Heatmap of the regression coefficients of the Cox regression model of the metabolic checkpoint genes. Coefficient greater than 0 indicates that the expression of metabolic checkpoint genes increases the risk of patient death, while a coefficient less than 0 indicates that the expression of metabolic checkpoint genes decreases the risk of patient death. * indicates a significant association between the metabolic checkpoint gene and cancer prognosis. (**D**) Ratio of good or poor survival-associated pairs of metabolic checkpoint gene and cancer. (**E**) Number of cancers with prognosis-associated metabolic checkpoint gene. (**F**) Number of metabolic checkpoint genes associated with survival of each cancer.

We collected a list of 26 metabolic checkpoint genes (MCGs) that have been previously validated [12], belong to three different metabolic landscapes (nutrient-sparse, metabolite-excessive, low-oxygen) in tumor microenvironments (Figure 1A). Interaction analysis revealed widespread interactions among these MCGs (Figure 1B). Then, 9585 patients from 33 cancer types in TCGA cohorts with clearly defined clinical information were selected (Table S1) and their transcriptomic data were integrated to assess the correlation of MCG expression (Figure S1) and the survival of TCGA cancer patients. To avoid the interference caused by age and gender, we used a multivariate Cox regression model to evaluate the correlation.

After eliminating the biases of age and gender, among the 26 MCGs, we observed 23 MCGs significantly affecting the survival of at least one type of cancer patient (Figure 1C). Of all combinations that were significantly correlated, we found that high expression of metabolic checkpoint genes more often leads to poor patient prognosis (Figure 1D). Among all MCGs, ADORA2A (adenosine A2a receptor) and XBP1 (X-box-binding protein 1) are significantly correlated with the survival of six types of cancer (Figure 1E), including increased risk of death in lung squamous cell carcinoma (HR = 1.42) and reduced risk of death in five other types of cancer patients (BRCA, HR = 0.53; HNSC, HR = 0.64; PAAD, HR = 0.47; SARC, HR = 0.62; SKCM, HR = 0.71). ADORA2A can also serve as a targetable immune checkpoint, and studies have shown that blocking ADORA2A can effectively treat refractory renal cell carcinoma [15]. XBP1 significantly reduced the risk of death in HNSC patients (HR = 0.71), which is observed in previous work [16]. In addition to HNSC, high XBP1 expression also significantly increased the risk of death in two types of cancer patients (LGG, HR = 1.82; UVM, HR = 24.24) and reduced the risk of death in three types of cancer patients (OV, HR = 0.52; PCPG, HR = 0.17; SKCM, HR = 0.64) (Figure 1C).

Considering individual cancers, the prognoses of eighteen cancers are significantly correlated with at least one MCG. Among those, kidney renal clear cell carcinoma (KIRC) and lower-grade glioma (LGG) have 13 prognosis-associated MCGs, the most among all cancers (Figure 1F). The high expressions of ACAT1 (HR = 0.49), AHR (HR = 0.71), ENO1 (HR = 0.69), GLUD1 (HR = 0.67), NFATC2 (HR = 0.66), PCK1 (HR = 0.54), and SLC16A4 (HR = 0.62) are significantly correlated with good prognosis in KIRC patients, while the high expression of ADORA2B (HR = 1.65), ARG1 (HR = 1.38), HIF1A (HR = 1.42), PVR (HR = 2.25), SLC1A5 (HR = 1.62), and TIGIT (HR = 1.49) are significantly correlated with poor prognosis in KIRC. Acetyl-CoA acetyltransferase 1 (ACAT1) has been shown to be downregulated in KIRC and overexpression of ACAT1 can inhibit the secretion of MMP7 in KIRC cells, thereby inhibiting tumor invasion [17]. Previous work revealed that high expression of glutamate dehydrogenase 1 (GLUD1) is detrimental to the survival of renal cancer cells in unfavorable nutritional environments (such as amino acid deficiency) and is significantly associated with good prognosis in KIRC patients [18]. High expression levels of adenosine A2b receptor (ADORA2B) and solute carrier family 1 member 5 (SLC1A5) were also correlated with poor prognosis in KIRC patients [19,20]. Further, the high expression of ACAT1 (HR = 0.44), GLUD1 (HR = 0.41), SLC38A1 (HR = 0.61), and SLC38A2 (HR = 0.63) are significantly correlated with good prognosis in LGG patients, while high expression of AHR (HR = 1.78), ENO1 (HR = 1.69), HK2 (HR = 3.56), MTOR (HR = 1.71), NFATC2 (HR = 2.16), SLC16A4 (HR = 2.92), SLC1A5 (HR = 3.00), SLC36A4 (HR = 1.67), and XBP1 (HR = 1.82) are correlated with poor prognosis in LGG patients. The expression of enolase 1 (ENO1) and hexokinase 2 (HK2) has been shown to play an important role in the occurrence and metastasis of glioma cells [21,22].

### 3.2. Extensive Impact of the Activities of Metabolic Pathways on Cancer Prognosis

**Figure 2:**
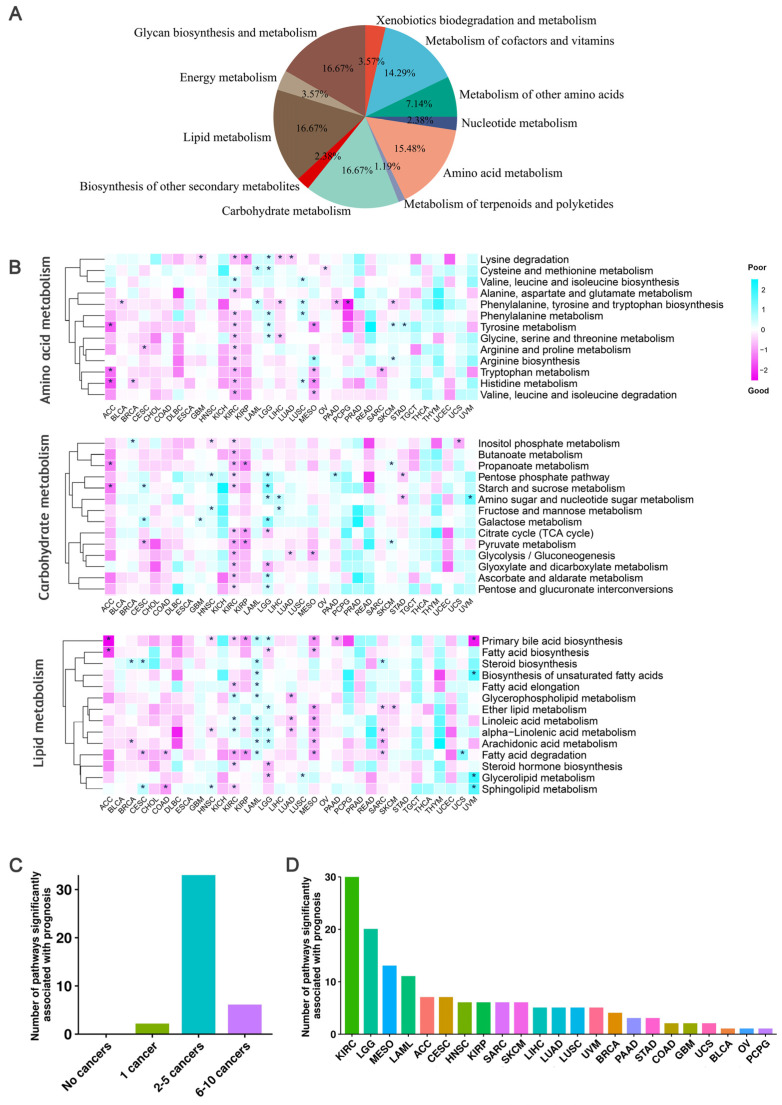
Correlation of 41 metabolic pathways from three primary categories and cancer survival. (**A**) A total of 84 metabolic pathways from 11 major metabolite-related subtypes defined by KEGG were used. (**B**) Heatmap of the regression coefficients of the Cox regression model of the three main metabolite-related pathways. A coefficient greater than 0 indicates that the metabolic pathway increases the risk of patient death, while a coefficient less than 0 indicates that the metabolic pathway decreases the risk of patient death. * indicates a significant association between the metabolic pathway and cancer prognosis. (**C**) Bar chart showing the total number of metabolic pathways significantly associated with a specific number of cancer prognoses in the three main metabolite-related pathways. The height of the bar represents the total number of metabolic pathways significantly associated with a specific number of cancer prognoses. (**D**) Statistics of the number of major metabolite-related metabolic pathways significantly associated with cancer prognosis in each cancer type.

To inspect the influence of metabolic pathways (MPs) in cancer prognosis, we collected 84 metabolic pathways from the KEGG [13] database, belonging to 11 major categories (Figure 2A, Tables S2 and S3), and calculated their activities using transcriptomic data (see Materials and Methods). Notably, the activities of all MPs in liver hepatocellular carcinoma (LIHC) were relatively high, comparing to other cancers (Figure S2, Table S4). We constructed a multivariable Cox regression model with the activities of 84 MPs, age, and gender information as independent variables in assessing the association between the activities of MPs and cancer prognosis.

We first inspected the impact of 41 pathways from three primary metabolic categories: amino acid metabolism, carbohydrate metabolism, and lipid metabolism. Results showed that most of these pathways are significantly correlated with the survival of at least one cancer (Figure 2B,C). For example, in the lipid metabolic pathway, the sub-pathway primary bile acid biosynthesis had the most significant impact on the prognosis of nine cancers. In pancreatic cancer, ursodeoxycholic acid (UDCA) has anticancer effects, while deoxycholic acid (DCA) and CDCA have procancer effects [23]. In our results, we observed that the activity of primary bile acid biosynthesis pathway is significantly correlated with the prognosis of pancreatic cancer (PAAD) patients (HR = 0.46). In addition, the activity of primary bile acid biosynthesis pathway is also significantly associated with good prognoses in adrenocortical carcinoma (ACC) (HR = 0.06), kidney renal papillary cell carcinoma (KIRP) (HR = 0.37), mesothelioma (MESO) (HR = 0.36), uveal melanoma (UVM) (HR = 0.11), HNSC (HR = 0.69), and KIRC (HR = 0.62), and with poor prognoses in LAML (HR = 1.89) and LGG (HR = 1.64). Several MPs are only significantly associated with the prognosis of special cancer. For example, alanine, aspartate, glutamate, and butanoate metabolism are only significantly correlated with the prognosis of KIRC.

Another 43 pathways from eight nonprimary metabolic categories including biosynthesis of other secondary metabolites, energy metabolism, glycan biosynthesis and metabolism, metabolism of cofactors and vitamins, metabolism of other amino acids, metabolism of terpenoids and polyketides, nucleotide metabolism, and xenobiotics biodegradation and metabolism were further inspected (Figure S3A). Results indicated the mannose type O-glycan biosynthesis in glycan biosynthesis and metabolism showed a significant association with the prognosis of seven cancers: with poor prognosis in sarcoma (SARC) (HR = 1.66), ACC (HR = 17.18), CESC (HR = 2.23), KIRC (HR = 1.47), MESO (HR = 4.78), and SKCM (HR = 1.43), and good prognosis in stomach adenocarcinoma (STAD) (HR = 0.59). Existing studies have shown that cancer cells often synthesize polysaccharides at different levels and that specific glycosylation patterns may be useful for tumor grading and prognosis [24]. Among all pathways, seven pathways showed significant association with prognosis in only one cancer (Figure S3B). For example, caffeine metabolism (HR = 0.60) was significantly associated with good prognosis of invasive breast carcinoma (BRCA), while glycosphingolipid biosynthesis-lacto and neolacto series (HR = 1.91) and vitamin B6 metabolism (HR = 1.70) were significantly associated with poor prognosis of BRCA. D-amino acid metabolism (HR = 0.49), glycosylphosphatidylinositol (GPI)-anchor biosynthesis (HR = 0.67), and riboflavin metabolism (HR = 0.11) were correlated with good prognosis in KIRC, lung squamous cell carcinoma (LUSC), and uveal melanoma (UVM), respectively. Mucin type O-glycan biosynthesis was only significantly associated with poor prognosis in head and neck squamous cell carcinoma (HNSC) (HR = 1.59). Mucin-type O-glycans are a class of glycans initiated with N-acetylgalactosamine (GalNAc) α-linked, and changes in their intracellular content have been described in various types of tumors, which may affect cancer prognosis [25].

An interesting result is that we observed the prognosis of KIRC and LGG have the strongest significant associations with the activities of all pathways (Figure 2D and Figure S3C). Considering KIRC has long been recognized as a metabolic disease due to abnormal accumulation of lipid droplets in the cytoplasm [26], and LGG is a group of primary brain tumors produced by supporting glial cells and characterized by mutations in isocitrate dehydrogenase (IDH) [27]. Overall, these MPs significantly associated with cancer prognosis may have potential applications in cancer prevention and treatment.

## 4. Discussion

Metabolic checkpoints regulate the immune response in cancer and immunotherapy by coordinating metabolic interactions between tumor cells and infiltrating immune cells. In this study, we systematically identified the survival and efficacy-related metabolic transcriptomes across multiple cancers based on TCGA datasets, which provided abundant and comparable cancer samples [28]. The expression of ADORA2A and XBP1 was found to have a significant impact on the survival of most cancer types. ADORA2A, the adenosine receptor of the A2A subtype, interacts with G protein family members to increase intracellular cAMP levels. In the tumor microenvironment, the expression of ADORA2A affects the function, differentiation, and number of CD8+ T cells [29]. The downregulation of ADORA2A expression using nanoparticles in HNSC patients increased T cell infiltration into tumors [30]. This is consistent with our observation that an increase in ADORA2A expression significantly reduces the risk of death in HNSC patients. X-box-binding protein 1 (XBP1) encodes a transcription factor that regulates MHC class II genes by binding to a promoter element referred to as an X box. The IRE1α-XBP1 pathway that XBP1 participates in plays a critical role in physiological and pathological environments, and its activity has a profound impact on disease progression and prognosis [31]. An increase in XBP1 expression is associated with a good prognosis in HNSC patients [32]. This is similar to our findings, which show that XBP1 significantly reduces the risk of death in HNSC patients (HR = 0.71).

Abnormal cancer metabolism, such as aerobic glycolysis and increased synthetic pathways, play important roles in tumor initiation, metastasis, drug resistance, and cancer stem cells [33]. As the main source of cellular energy, glucose metabolism is also a key source of carbon for cancer cell metabolism [34]. Glucose uptake also restricts T cell activation [35]. The expression profile of carbohydrate pathway-related genes in cancer cells is related to the tumor cell dissemination pathway, metastasis mode, and prognosis of colorectal cancer [36]. Fatty acids not only have a structural role, but also act as secondary messengers (DAG and IP3), regulating multiple physiological processes, including cell signaling, ultimately leading to the regulation of T cell function [37]. Therefore, fatty acid synthesis is essential for cell response and proliferation. Our research found that the activation levels of multiple pathways related to the amino acid metabolism and carbohydrate metabolism are significantly associated with patient survival. Lipid levels can also serve as predictors of breast cancer risk and prognosis, with an increase in levels reducing the risk of death in breast cancer patients [38]. We found that the levels of most pathways in the lipid metabolism-related metabolic pathway reduce the risk of death in BRCA patients, although not all have statistically significant associations. In addition, research has found that lipid metabolism is related to the prognosis and incidence of colon cancer [39]. In our study, we found that the levels of two lipid metabolism-related pathways (fatty acid degradation, steroid biosynthesis) were significantly associated with the survival outcomes of colon adenocarcinoma (COAD) patients, and an increase in their activation levels significantly reduced the risk of death in COAD patients.

## 5. Conclusions

Overall, through integrating the expression and clinical outcomes at pan-cancer level, our research revealed that metabolic checkpoint genes and pathways are significantly associated with cancer prognosis, suggesting potential metabolic transcriptomic markers in evaluating the cancer prognosis and treatment.

## Data Availability

Not applicable.

## Supplementary Materials

The following supporting information can be downloaded at: https://www.mdpi.com/article/10.3390/biology12081129/s1. Figure S1. Expression matrix of 26 metabolic checkpoint genes in 33 cancer types. Expression value was represented by log_2_ (x + 1), while x indicates the RSEM normalized counts of each gene. The average gene expression of all samples in each cancer was scaled by rows. Red represents high expression and blue represents low expression. Figure S2. Activity score of 84 metabolic pathways from 11 major categories in 33 cancer types. Each cell was represented by the average original ssGSEA scores of all samples in each cancer. Red indicates a high level of pathway activity and blue indicates the low activity. Figure S3. Correlation of 43 pathways from eight nonprimary metabolic categories and cancer survival. A. Heatmap of the regression coefficients of the Cox regression model for nonprimary metabolite related pathways. Positive coefficients indicate an increased risk of patient mortality associated with the metabolic pathway, while negative coefficients indicate decreased risk. * indicates a significant association between the metabolic pathway and cancer prognosis. B. The statistics for significant combinations of nonprimary metabolite related pathways associated with a specific number of cancer cases. The height of the bar represents the total number of metabolic pathways significantly associated with the prognosis of a specific number of cancers. C. Number of nonprimary metabolite-related metabolic pathways significantly associated with prognosis for each cancer type. Table S1. Summary of cancer samples in TCGA cohort. Table S2. Eighty-four metabolic pathways from 11 major categories. Table S3. Gene list of 84 metabolic pathways. Table S4. The ssGSEA scores of 84 metabolic pathways in TCGA cohort.
