# Supplementary figures and images for: Pan-Cancer Identification of Prognostic-Associated Metabolic Pathways
Source: Biology (Basel). 2023 Aug 14;12(8):1129. doi: 10.3390/biology12081129 (PMC10452188; doi:10.3390/biology12081129)

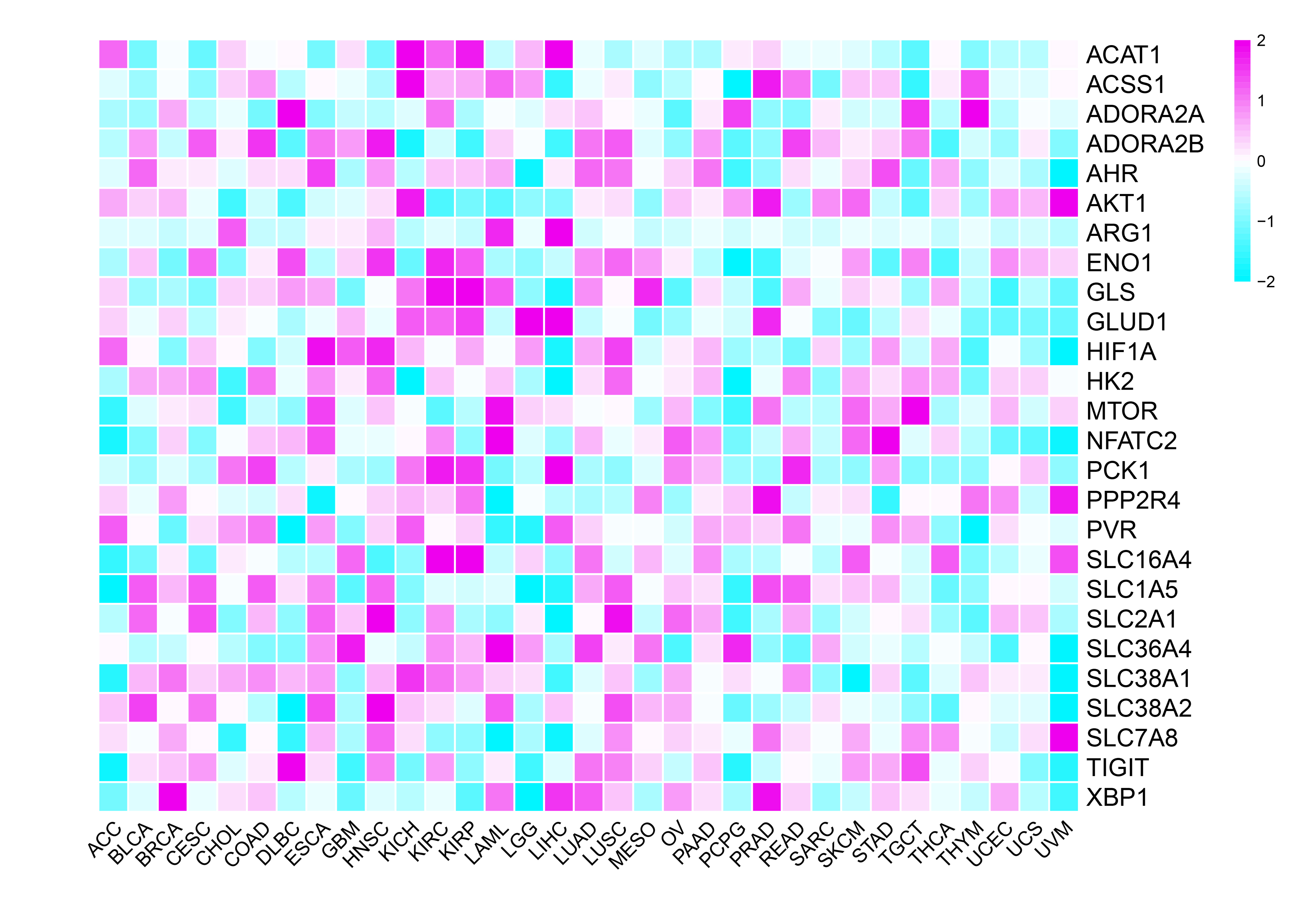

Supplement: Supplementary file 1 [file biology-12-01129-s001.zip › Figure S1.jpg]

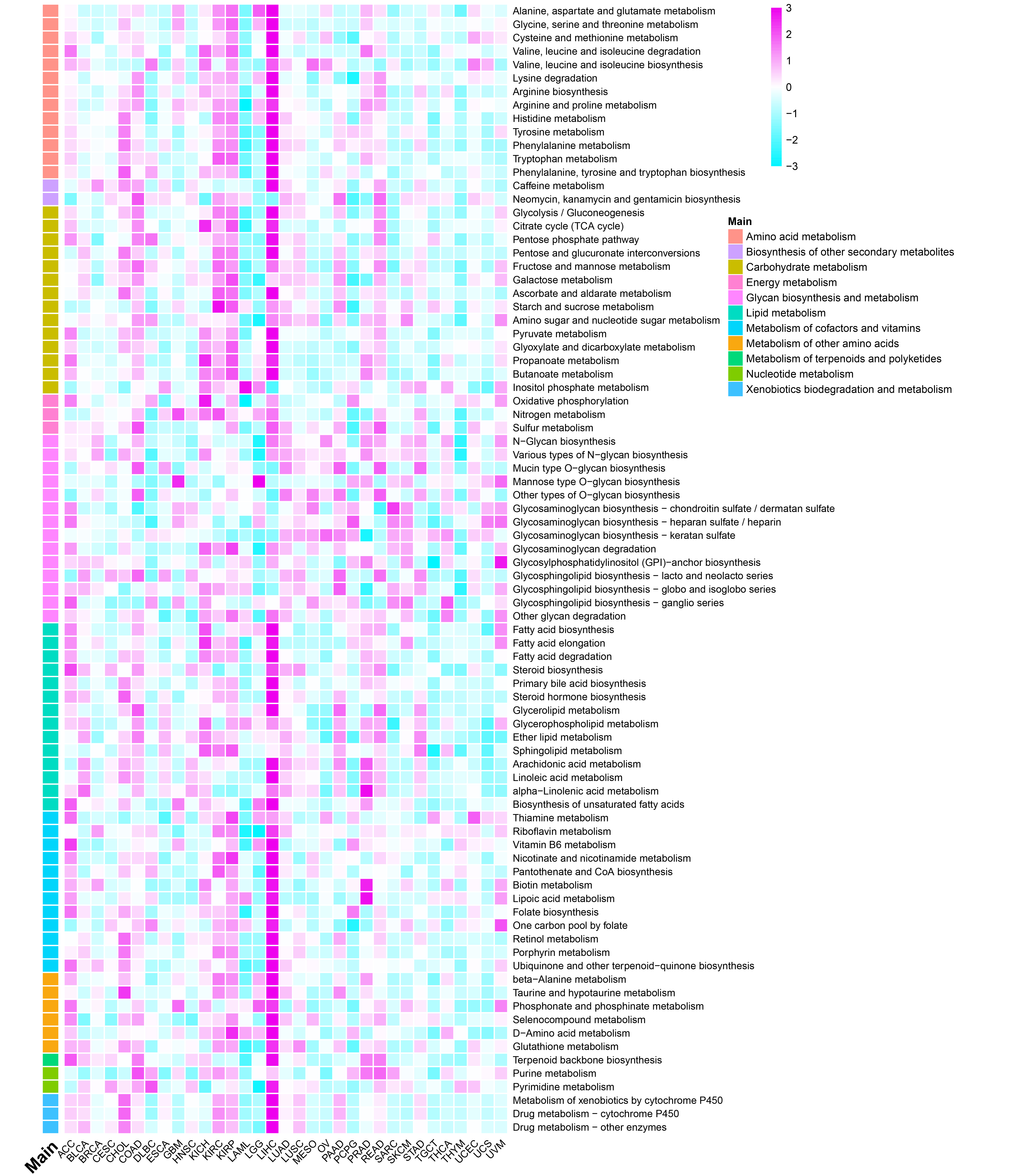

Supplement: Supplementary file 1 [file biology-12-01129-s001.zip › Figure S2.jpg]

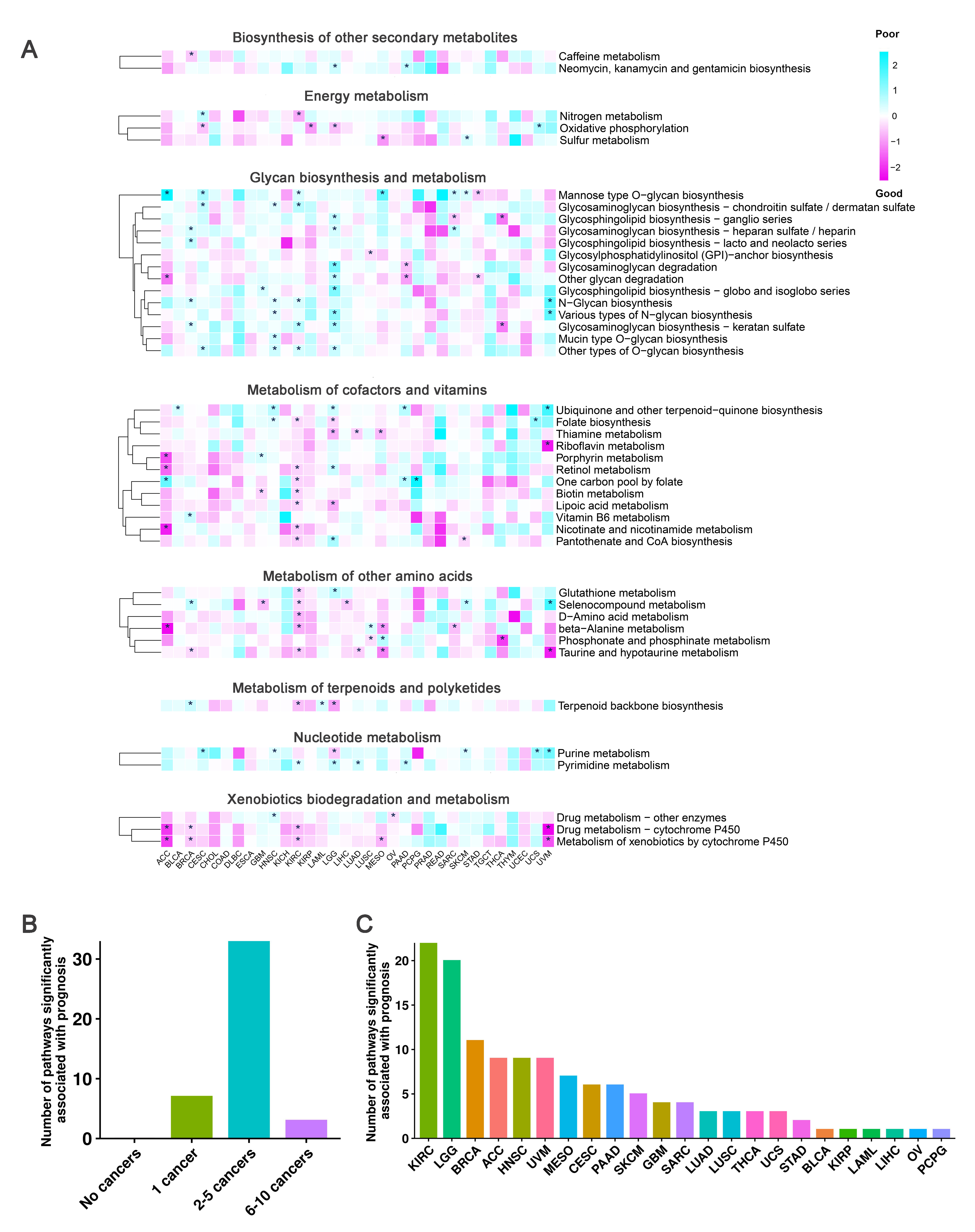

Supplement: Supplementary file 1 [file biology-12-01129-s001.zip › Figure S3.jpg]
